# The psychostimulant drug, fenethylline (captagon): Health risks, addiction and the global impact of illicit trade

**DOI:** 10.1016/j.dadr.2025.100323

**Published:** 2025-03-01

**Authors:** Matthew Chidozie Ogwu, Matěj Malík, Pavel Tlustoš, Jiří Patočka

**Affiliations:** a Goodnight Family Department of Sustainable Development, Appalachian State University, 212 Living Learning Center, 305 Bodenheimer Drive, Boone, NC 28608, United States; b Department of Agroenvironmental Chemistry and Plant Nutrition, Faculty of Agrobiology, Food and Natural Resources, Czech University of Life Sciences Prague, Kamýcká 129, Suchdol, Praha 165 00, Czech Republic; c Department of Radiology, Toxicology and Civil Protection, Faculty of Health and Social Studies, University of South Bohemia, J. Boreckého 1167/27, České Budějovice 370 11, Czech Republic

**Keywords:** Psychostimulant, Addiction, Withdrawal symptoms, Illicit trade, Global trafficking, Public health

## Abstract

Fenethylline (street name, captagon) is a synthetic amphetamine-type stimulant that is emerging as a significant public health and security concern, particularly in the Middle East. This systematic review synthesizes original research articles, epidemiological studies, systematic reviews, policy analyses, and case reports to provide a comprehensive analysis of fenethylline’s health impacts, addiction potential, and dynamics of illicit trade. Initially developed for therapeutic use, fenethylline illicit production and use have escalated, raising concern about its physiological, psychological, and socio-economic impacts. This stimulant profoundly affects the central nervous system, enhancing wakefulness, concentration, and physical stamina while inducing euphoria. These effects come at the cost of serious adverse health outcomes, particularly with prolonged or heavy use, including cardiovascular complications, neurological damage, and addiction. The dependence-forming nature of captagon contributes to escalating substance use disorders, impacting healthcare systems. Beyond its biomedical implications, fenethylline trafficking has become a global issue, with supply chains deeply intertwined with politically unstable regions where illicit economies thrive. The geopolitical dimensions of captagon’s trade amplify its global security threat, influencing international relations and regional stability. This paper underscores the urgent need for systematic data collection and coordinated efforts to regulate illicit fenethylline production and distribution. Strategies such as improved surveillance, public health interventions, and international cooperation are essential to mitigate its escalating risks. Addressing this issue requires a multidisciplinary approach, integrating public health, law enforcement, and policy development to curb its impact on global health and security.

## Highlights

• Fenethylline (captagon), once therapeutic, is now an illicit public health threat.
• Misuse of captagon leads to severe health risks, including addiction.
• Captagon production and trafficking are linked to organized crime in conflict zones.
• An integrated approach is necessary to combat the misuse of captagon.
• More research is needed to understand the effects of captagon use.

## 1 Introduction

**Fig. 1:**
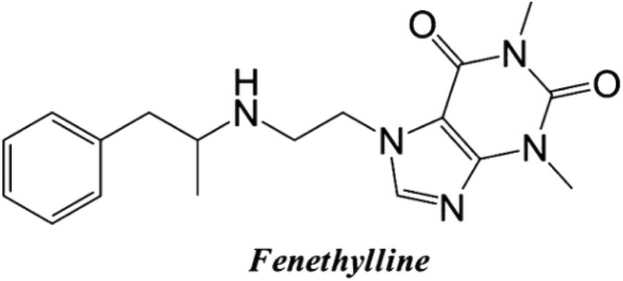
Chemical structure of fenethylline (captagon).

Captagon (also called capatgon) is the street name for the drug fenethylline (Fig. 1), also known as amfetamine-ethyl-theophylline or amfetyline (IUPAC name: (R,S)-1,3-dimehtyl-7-[2-(1-phenylpropan-2-ylamino) ethyl]purine-2,6-dione; (Katselou et al., 2016, Wu et al., 2019). According to Wu et al. (2019), fenethylline is a synthetic psychostimulant that acts as a prodrug of amphetamine and theophylline, substances with stimulating effects on the central nervous system (CNS). It was initially developed in the 1960s under names like Biocapton and Fitton to treat conditions such as hyperactivity, Attention-Deficit-Hyperactivity-Disorder (ADHD), narcolepsy, and depression, particularly in children (Katselou et al., 2016). The combined effects of amphetamine and theophylline produce effects such as increased energy, improved mood, and suppressed hunger (Al-Imam et al., 2017).

Initially, fenethylline was considered relatively safe for medical use, but its addictive potential soon became evident. After the cessation of legal production in 1986, the drug was banned, and illicitly manufactured fenethylline began to circulate, particularly in regions like South-Eastern Europe and parts of Asia (Fong et al., 2024). Currently, captagon is exclusively illicitly produced, as no legitimate pharmaceutical companies manufacture fenethylline for medical use. Eventually, the drug, known in the Middle East as 'chemical courage,' made its way into the region, particularly Syria, but also remained in Europe and was reported to be used by the Paris rioters in 2015 (Pergolizzi et al., 2024). According to Caesar (2024), fueled by the prolonged civil war, Syria has become a central hub for illicit captagon manufacturing, with production facilities operating in various regions. The drug is then trafficked through complex networks to neighboring countries and beyond, contributing to a multi-billion-dollar black market. Millions of captagon tablets are seized each year in Arab countries, accounting for nearly one-third of the world's amphetamine seizures annually (Dabbagh and Rawson, 2019, Kravitz and Nichols, 2016). The illicit use of the drug is especially problematic in countries like Jordan (Alabdalla, 2005), and reports indicate that its prolonged or heavy use is linked to serious health problems, including myocardial infarction (Ulucay et al., 2012) and cardiomyopathy (Elasfar et al., 2014). Moreover, the illicit captagon trade has been linked to terrorist organizations like the Islamic State of Iraq and Syria (ISIS), where it is used to fund their activities and enhance the performance of fighters in conflict zones (Al-Imam and AbdulMajeed, 2017). This amphetamine-type stimulant is known to reduce the need for rest and sleep, which is why it has been favored by militias and terrorists in war-torn regions (Al-Imam et al., 2017, Kravitz and Nichols, 2016). The widespread illicit production and distribution of captagon in war zones have drawn international attention due to the drug's connections to terrorism and regional instability. Efforts by pharmacologists, toxicologists, and forensic pathologists to monitor and curb the illicit use of captagon have been ongoing, but the situation remains dire, especially in the Middle East (Bamofleh et al., 2017, Laniel, 2017, Wu et al., 2019). International initiatives, including those by the World Health Organization (WHO), highlight the need for comprehensive action to control drug trafficking and mitigate the public health crisis stemming from captagon (Al-Imam et al., 2017, Mohaddes Ardabili et al., 2022).

This review evaluated existing data from forensic reports, scientific studies, and law enforcement records to assess the biomedical effects of fenethylline, its role in substance abuse patterns, and its impact on public health and security. Sources were systematically retrieved from databases such as Web of Science, PubMed, Scopus, and Google Scholar, covering clinical research, epidemiological data, and policy discussions. Building upon this foundation, the review provided a comprehensive overview of captagon, tracing its evolution from a legitimate pharmaceutical drug to a purely illicit psychostimulant. It examined the severe health risks associated with its misuse, including its effects on the CNS, cardiovascular diseases, and its broader contribution to the public health crisis, particularly in the Middle East. Additionally, the review highlighted captagon's role in exacerbating social issues, such as its connection to criminal and terrorist organizations in conflict zones. Examining the health and social ramifications of illicit captagon use, as well as the necessity of transnational cooperation, was essential to curb the illegal production, distribution, and usage of this stimulant. The overarching objective was to foster a clearer understanding of captagon's global impact and explore potential measures—such as policy interventions and scientific research—to mitigate the growing threat posed by this drug (Al-Imam et al., 2017, Keefe, 2016).

## 2 Chemical composition and pharmacology of captagon

### 2.1 Mind and body effects of captagon

Captagon (fenethylline) was originally developed as a pharmaceutical drug intended to treat disorders such as ADHD and narcolepsy (Kristen et al., 1986, Sevarino and Farrell, 2023). The drug is composed of amphetamine, which provides stimulant effects, and theophylline, which acts as a bronchodilator (Jilani et al., 2023, Martin and Le, 2023). Captagon's pharmacological action likely primarily targets neurotransmitters in the brain, particularly dopamine and norepinephrine, which play crucial roles in regulating motor and limbic functions, mood regulation, arousal, and cognitive function (Rothman et al., 2001). Once ingested, captagon has a range of physiological and psychological effects, depending on dosage, route of administration, and individual sensitivity. Among its most notable effects are presented in Table 1.

**Table 1:** Physiological and psychological effects of captagon.

**Effect**	**Health Impacts**	**References**

Euphoria	Induces intense feelings of euphoria, pleasure, and well-being, leading to a heightened sense of happiness and satisfaction.	Ciucă Anghel et al. (2023)
Increased Alertness and Energy	Acts as a CNS stimulant, increasing alertness, wakefulness, and energy levels, making users feel more awake and mentally sharp.	Wu et al. (2019)
Enhanced Cognitive Function	Improves cognitive functions such as memory, concentration, and attention span, enhancing mental clarity and performance.	Schifano et al. (2023)
Improved Mood	Elevates mood and enhances mood stability, leading to a more positive, confident, and sociable demeanor.	Katselou et al. (2016)
Suppressed Appetite	Suppresses appetite, reducing feelings of hunger and decreased food intake, potentially contributing to weight loss.	Katselou et al. (2016)
Increased Physical Stamina	Boosts physical stamina and endurance, allowing users to engage in prolonged physical activity without fatigue.	Ciucă Anghel et al. (2023)
Heightened Sensory Perception	Enhances sensory perception, increasing sensitivity to visual, auditory, and tactile stimuli, making colors more vibrant and sensations more intense.	Pergolizzi et al. (2024)
Tachycardia and Hypertension	Increases heart rate (tachycardia) and blood pressure (hypertension), posing cardiovascular risks, especially for those with pre-existing conditions.	Mathot (2018)
Dilated Pupils	Causes pupil dilation (mydriasis), often leading to changes in visual perception.	Bollu and Kaur (2019)
Insomnia and Restlessness	Disrupts sleep patterns, causing insomnia, restlessness, agitation, and anxiety, especially during the drug's comedown phase.	Bollu and Kaur (2019)
Psychological Effects	Induces psychological symptoms such as agitation, paranoia, hallucinations, and psychosis, particularly at higher doses or with chronic use.	Wu et al. (2019)

Some of the risks associated with captagon's stimulant effects include addiction, while cardiovascular complications, and psychological side effects may result after prolonged use (Desoky et al., 2011, Murphy, 2012). Furthermore, the misuse of captagon can have serious consequences for both physical and mental health (Alghamdi et al., 2016). Therefore, its recreational use is highly discouraged, and regulations should ensure access to the drug remains strictly controlled under medical supervision. Fenethylline is banned worldwide, and no legitimate pharmaceutical company produces it (Mohaddes Ardabili et al., 2022). Medical use has been discontinued in many countries due to concerns about its potential for misuse and addiction (Pergolizzi et al., 2024). The drug is now exclusively produced clandestinely in illicit laboratories, particularly in regions of the Middle East, where it has been linked to funding recreational use, conflicts, and terrorist activities. The original, legally manufactured captagon was a controlled pharmaceutical drug designed for therapeutic use with predictable effects and safety standards. In contrast, illicit captagon is an unregulated substance with varying and dangerous compositions, posing severe public health risks and fueling global trafficking networks. The differences between the originally legal pharmaceutical captagon and the current illicitly produced ones are highlighted in Table 2.

**Table 2:** Differences between the originally legal pharmaceutical captagon and the current illicitly produced ones.

**Original captagon (Fenethylline)**	**Illicit captagon**

Contains fenethylline, a molecule containing amphetamine and theophylline (sometimes as a combination product).	Typically, lacks fenethylline and is composed of amphetamines, methamphetamines, caffeine, and other adulterants.
Pharmaceutical-grade with controlled dosages.	It varies widely, often mixed with harmful adulterants and impurities.
Produced under-regulated pharmaceutical conditions with known safety profiles.	Manufactured clandestinely in unregulated environments with no quality control.
Balanced stimulant effects due to theophylline moderating amphetamine’s potency.	Amplified stimulant effects due to high amphetamine/methamphetamine content, increasing risks of severe side effects.
Relatively predictable side effects with known dosages.	Unpredictable health risks, including cardiovascular issues (e.g., hypertension, arrhythmias), neurological damage, and toxicity from adulterants.
Initially prescribed for medical conditions like ADHD and narcolepsy, later banned globally due to misuse.	Entirely illegal and linked to the underground drug trade.
Therapeutic doses are prescribed under medical supervision.	Abused recreationally, often consumed in highdoses or mixed with other substances.
Limited to misuse before its ban; controlled as a prescription drug.	Fuels organized crime, socio-political instability, and public health crises, particularly in the Middle East and Europe.

### 2.2 Components of captagon

**Fig. 2:**
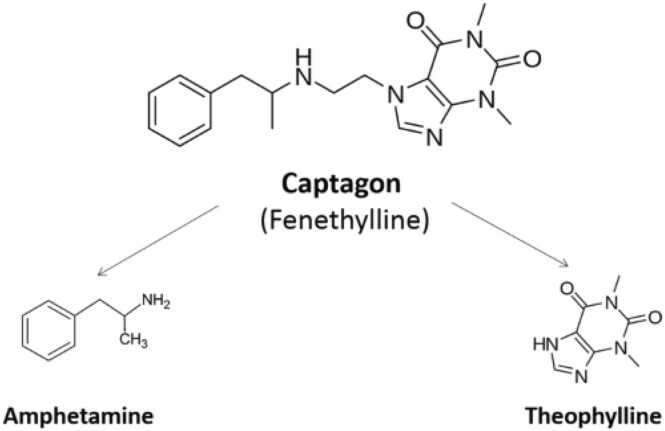
Chemical composition of captagon.

Captagon is a drug composed of two primary components: amphetamine and theophylline (Fig. 2), each contributing to captagon's pharmacological effects and properties, though they have distinct characteristics and mechanisms of action.

#### 2.2.1 Amphetamine

Amphetamine belongs to the class of phenethylamine compounds and has a structure similar to other sympathomimetic amines (Pires et al., 2023). It is a potent CNS stimulant that increases the release of neurotransmitters such as dopamine, norepinephrine, and serotonin in the brain (Heal et al., 2013, Nieddu et al., 2023). This leads to heightened alertness, enhanced focus, elevated mood, suppressed appetite, increased energy, and euphoria. While specific amphetamine derivatives, such as dextroamphetamine and lisdexamfetamine, are approved in certain countries for medicatABLE 3 l treatment of ADHD and narcolepsy, fenethylline (marketed as captagon) was similarly prescribed in the past but has since been banned due to widespread misuse (Castells et al., 2018, Katselou et al., 2016). However, due to the growing rate of illicit use and addiction, its medical use is highly regulated (Costa et al., 2022). Amphetamine can be synthesized through various chemical routes, often involving the precursor benzyl methyl ketone (BMK) and reagents like methylamine or ammonia (Di Giovanni et al., 2012). Amphetamine is primarily metabolized in the liver by enzymes such as cytochrome P450, producing metabolites like norephedrine. It is eliminated through urine, where both unchanged amphetamine and its metabolites are excreted (Kaleta, 2020). Common side effects of amphetamine include increased heart rate, elevated blood pressure, insomnia, agitation, and the potential for use disorder and addiction (Martin and Le, 2023).

#### 2.2.2 Theophylline

Theophylline belongs to the class of methylxanthine compounds and is structurally related to caffeine (Monteiro et al., 2016). It is a bronchodilator and vasodilator often used to treat respiratory conditions like asthma and chronic obstructive pulmonary disease (COPD) (Janitschke et al., 2020). It helps alleviate symptoms such as wheezing, shortness of breath, and chest tightness. Theophylline is usually synthesized from xanthine derivatives via methylation (Abu-Hashem et al., 2024). Theophylline is metabolized in the liver, primarily through the enzyme cytochrome P450 (CYP1A2). It is converted into metabolites like 1-methyluric acid and is excreted in the urine (Arnaud, 2011). Common side effects of theophylline include nausea, vomiting, headache, insomnia, palpitations, and gastrointestinal disturbances (Singh et al., 2018). At high doses, it can cause more severe adverse effects such as seizures, cardiac arrhythmias, and toxicity (Goh et al., 2023).

Fenethylline, containing elements of the structures of amphetamine and theophylline, produces both amphetamine-like and theophylline-like effects (Fong et al., 2024). Due to their potential for misuse and adverse effects, both amphetamine and theophylline are regulated substances with controlled medical use and restricted availability (Docherty and Alsufyani, 2021).

### 2.3 Mechanism of action of captagon

**Fig. 3:**
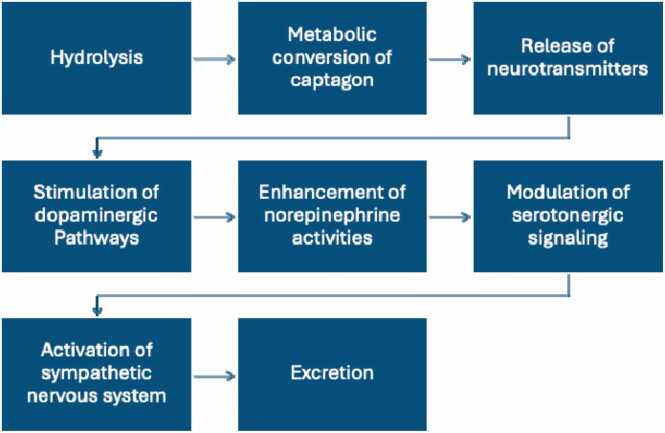
The processes involved in captagon's mechanism of action.

The mechanism of action of captagon involves its metabolic conversion in the body to produce active metabolites, primarily amphetamine. Some of the mechanisms involved are included (Fig. 3):

**Hydrolysis:** Fenethylline, the major compound in captagon, undergoes enzymatic hydrolysis in the gastrointestinal tract, primarily in the liver, to release its active metabolites (Lucchetti et al., 2021, Wenthur et al., 2017). This hydrolysis process involves enzymatic cleavage of the ester bond in fenethylline, resulting in the formation of amphetamine and theophylline.

**Metabolic conversion:** Fenethylline (captagon) is metabolized in the body through the cytochrome P450 (CYP450) enzyme system when taken orally, which generates approximately 24.5 % amphetamine and 13.7 % theophylline (Wu et al., 2019). The study by Katselou et al. (2016) revealed that fenethylline undergoes a complex metabolic process involving multiple stages. Initially, it is hydroxylated (oxidized), producing two primary metabolites: oxyethyltheophylline and amphetamine. Oxyethyltheophylline is metabolized into theophylline, with only a tiny portion of theophylline excreted unchanged (Ellison et al., 1970). Additional intermediate metabolites include 1,3-dimethyluric acid, 1-methyluric acid, and 3-methylxanthine, which contribute to the pharmacodynamic effects of fenethylline (Goenechea and Brzezinka, 1984). An alternative metabolic pathway suggests the formation of 7-aminoethyltheophylline and phenylacetone, with subsequent metabolites such as hippuric acid and possibly theophylline undergoing further transformations (Ellison et al., 1970, Goenechea and Brzezinka, 1984). Studies by Yoshimura et al. (1988) revealed six metabolites in the urine of rats and human volunteers following oral administration, including amphetamine, p-hydroxyamphetamine, acetylaminoethyl theophylline, 7-aminoethyltheophylline, hydroxyethyl theophylline, and carboxymethyl theophylline. The metabolism involves oxidative cleavage at two distinct sites, forming aminoethyl theophylline and amphetamine. Among the major metabolites, amphetamine remains detectable in human urine for 24–48 hours, whereas carboxymethyl theophylline is present only in trace amounts after the same period (Katselou et al., 2016).

**Release of neurotransmitters:** Amphetamine, one of the primary metabolites of captagon, undergoes further metabolism in the liver by various cytochrome P450 enzymes, particularly CYP2D6 and CYP3A4 (Zhao et al., 2021). These enzymes catalyze the conversion of amphetamine into its active enantiomers: d-amphetamine (dextroamphetamine or (+)-amphetamine) and l-amphetamine (levoamphetamine or (–)-amphetamine). Specifically, d-amphetamine is more potent in stimulating the central nervous system, while l-amphetamine exhibits relatively weaker stimulant effects but contributes to cardiovascular activity. This clarification aligns with the specific isomeric forms relevant to fenethylline's metabolism and their contributions to its pharmacological profile. The pharmacodynamics of fenethylline involve its action on monoamine transporters, which are crucial for regulating neurotransmitters such as dopamine, serotonin, and norepinephrine (Ferrucci et al., 2019). These neurotransmitters play significant roles in mood regulation, alertness, and overall cognitive function (Katselou et al., 2016). The presence of the xanthine component, derived from theophylline, adds another layer of complexity, as it can influence the stimulant effects by modulating adenosine receptors, which are known to affect arousal and sedation (Wenthur et al., 2017).

**Stimulation of dopaminergic pathways:** The release of dopamine in the mesolimbic and mesocortical pathways of the brain's reward system is thought to underlie the reinforcing and euphoric effects of amphetamine (Adinoff, 2004, Feltenstein and See, 2008). This dopaminergic stimulation contributes to the feelings of pleasure, reward, and motivation associated with captagon use.

**Enhancement of norepinephrine activities:** Amphetamine also increases the release and inhibits the reuptake of norepinephrine, leading to heightened arousal, increased alertness, and enhanced cognitive function (Sofuoglu and Sewell, 2009)These effects contribute to captagon's stimulant properties, including increased energy levels and improved focus and attention.

**Modulation of serotonergic signaling:** In addition to its effects on dopamine and norepinephrine, amphetamine can modulate serotonin neurotransmission in the brain (Faraone, 2018). This modulation may influence mood regulation, appetite suppression, and other physiological processes associated with captagon use.

**Activation of the sympathetic nervous system:** The release of dopamine and norepinephrine by amphetamine also activates the sympathetic nervous system, leading to adverse physiological responses such as increased heart rate, elevated blood pressure, and bronchodilation (Gordan et al., 2015, Rusyniak, 2011).

**Excretion:** Once metabolized, the active metabolites of captagon, including amphetamine and its derivatives, are excreted from the body primarily through renal clearance (Wagner et al., 2017). They are filtered by the kidneys and eliminated in the urine in their metabolized form. The elimination half-life of captagon and its metabolites varies depending on factors such as dose, route of administration, and individual metabolic rate. It is rapidly absorbed, reaching peak plasma levels within about one hour, and has an estimated half-life of approximately 1.3 hours.

## 3 Addiction consequences and withdrawal symptoms of captagon

Captagon has been associated with a range of psychological and physiological effects that complicate its use and withdrawal. Addiction to captagon is characterized by compulsive use despite adverse consequences, a hallmark of substance use disorders (Katselou et al., 2016, Pergolizzi et al., 2024). The physiological effects of captagon misuse extend beyond psychological disturbances; studies have indicated that prolonged use can lead to significant organ dysfunction, particularly affecting liver and renal functions (Katselou et al., 2016, Pergolizzi et al., 2024). Amphetamine is the primary contributor to fenethylline's addictive properties, while theophylline is thought to counteract behavioral sensitization caused by repeated amphetamine use (Wu et al., 2019). This interaction may account for fenethylline's lower addictive potential compared to amphetamine alone. Logan et al. (2023) reported that recreational doses of amphetamines usually range from 50 to 100 mg per use and are associated with significant stimulation, including heightened excitement, euphoria, rapid speech, racing thoughts, excessive motor activity, increased heart rate, and elevated blood pressure. Long-term use can lead to psychological symptoms such as paranoia, panic attacks, and delusional thinking. These effects are often more severe when the drug is taken intravenously or through smoking (Logan et al., 2023).

Due to adverse negative effects and non-medical use concerns, fenethylline was classified as a Schedule II substance under the UN Convention on Psychotropic Substances 1971 in 1986, ceasing therapeutic use (EMCDDA, 2018). Nevertheless, clandestine production in home labs has resulted in counterfeit captagon tablets containing various substances, including impurities. While contemporary tablets may bear a similar logo, they often lack fenethylline (Katselou et al., 2016). Captagon tablets typically lack this original compound and are instead composed of amphetamines, methamphetamines, caffeine, and other adulterants. These counterfeit versions are produced illicitly, with their chemical composition and general characteristics varying significantly depending on the production location and available materials. The counterfeit production of captagon is growing in regions such as the Middle East, Europe, and North Africa (Fong et al., 2024). Withdrawal symptoms from captagon can be particularly challenging, as they encompass both psychological and physical dimensions. The psychological withdrawal symptoms can be profound, with some individuals exhibiting violent behavior and severe mood swings, which complicates treatment and recovery efforts (Pergolizzi et al., 2024). Additionally, the presence of co-occurring disorders, such as opioid use disorder, can further complicate the withdrawal process and the overall treatment landscape for individuals addicted to captagon (Katselou et al., 2016, Pergolizzi et al., 2024). The socio-demographic characteristics of captagon users also play a crucial role in understanding the addiction consequences. Many users are young, with a significant percentage starting their substance use during adolescence, which can lead to long-term psychological and social issues (Alshenguity et al., 2023). The impact of captagon addiction is not only on an individual but extends to familial and societal levels, with high rates of unemployment and social dysfunction reported among users (Alshenguity et al., 2023).

### 3.1 Development of tolerance for captagon

Captagon's use is primarily motivated by economic, health, or social factors. Economic incentives prevail, especially for enhanced work or study performance (Kravitz and Nichols, 2016, Wazaify et al., 2024). Individuals may experiment with captagon out of curiosity, to escape family issues, or due to peer influence during social gatherings (Khalil et al., 2022). Some mention weight loss as a motivation (Wazaify et al., 2024). While specific case studies directly linking captagon use to weight loss are scarce, the drug's appetite-suppressing properties have been broadly suggested as a correlation or side effect (Katselou et al., 2016, Katz, 2022). For instance, a study involving Jordanian university students indicated that individuals misusing captagon experienced reduced appetite and notable weight loss as a secondary effect of the drug's stimulant properties (Al Omari et al., 2022). Additionally, it has been observed that specific demographics, particularly young individuals, may exploit captagon's stimulant effects for weight management purposes, further highlighting its misuse in the context of dieting and body image (Al-Imam et al., 2017). Although captagon may exhibit appetite-suppressing properties that some individuals exploit for weight loss, the associated health risks are profound and multifaceted (Katselou et al., 2016).

The type and source of illicit captagon pills play a crucial role, with costlier varieties perceived as purer. While cheaper pills with two crescents are widely available, pricier options with specific engravings, reportedly imported from Israel, are known for intensified and longer-lasting effects (Kravitz and Nichols, 2016, Wazaify et al., 2024). Users may escalate from smaller quantities to high daily doses, developing tolerance over time (Thomas, 2013). Despite varying prices, participants agree on captagon's relative affordability, contributing to its widespread use among youth (Alabdulla et al., 2022).

### 3.2 Use disorder and addiction to captagon

Captagon's highly addictive nature is reflected in increased energy levels, severe headaches, mood swings, decreased appetite, rapid breathing, dilated pupils, and anxiety (Abazid, 2022). Short-term use poses risks of amphetamine-induced psychosis and acute hearing loss, while chronic use links to severe complications like myocardial infarction (heart attack), hemorrhagic central retinal vein occlusion, suicidal ideation, and cognitive decline (Dabbagh and Rawson, 2019, Ulucay et al., 2012). The work of Gokdemir and Giden (2019) highlights the case of a 23-year-old male who experienced an acute inferior myocardial infarction after ingesting 2–3 captagon tablets. The patient presented with severe chest pain, shortness of breath, and ST-segment elevation on an electrocardiogram. Despite normal coronary angiographic findings, the heart attack was attributed to coronary vasospasm caused by captagon use. This case underscores the potential of captagon to induce serious cardiovascular events, even in individuals without pre-existing cardiac risk factors (Gokdemir and Giden, 2019). Reported side effects of captagon vary, with nausea, paranoia, distrust, and negative impacts on teeth health with prolonged or heavy use rather than isolated exposure (Wazaify et al., 2024). Signs of addiction include persistent drug cravings, increased energy despite inadequate sleep, blurred vision, and drastic weight loss (El Khoury, 2018, Katselou et al., 2016).

### 3.3 Withdrawal symptoms and challenges

Long-term use leads to withdrawal symptoms, contrary to captagon's initial effects (Kristen et al., 1986). Captagon withdrawal symptoms encompass psychomotor agitation or retardation, laziness, sleepiness, irritability, depression, aches, fatigue, unpleasant dreams, increased appetite, insomnia or hypersomnia, and social withdrawal (Abazid, 2022, Yasin et al., 2020). Although not life-threatening, these symptoms cause significant discomfort (White et al., 2021). Attempts to quit often fail due to mentioned intense withdrawal symptoms and drug cravings (Al-Imam et al., 2017). The illicit use of captagon can severely impact the body, necessitating a comprehensive program for successful rehabilitation (Steenkamp, 2024). A case involving a middle-aged man with iatrogenic opioid use disorder highlighted the dangers of using captagon to manage opioid withdrawal pain, emphasizing the need for careful counseling and follow-up when prescribing opioids (Hamdan et al., 2021). While captagon intoxication is managed similarly to other stimulant types, further studies are essential to develop substance-specific management guidelines (Yasin et al., 2020).

Saudi medical literature pays scant attention to female illegal substance use, with studies predominantly focusing on men. This bias may stem from specialized substance use disorder treatment centers, excluding women who seek assistance at psychiatric hospitals (Wazaify et al., 2024).

## 4 Production and trafficking of captagon

Originally, it was first synthesized by Degussa AG, a German pharmaceutical company, in 1961. Captagon continues to garner global attention due to its widespread illicit production and trafficking (Al-Imam et al., 2017, Steenkamp, 2024). The production of captagon involves clandestine laboratories operating outside legal frameworks, often in remote or inaccessible locations, to evade law enforcement scrutiny (Al-Obaidi and Fletcher, 2014). The illicit manufacturing of captagon involves obtaining precursor chemicals, such as ephedrine or pseudoephedrine, which are necessary for the synthesis of amphetamine (Citaristi, 2022). These precursor chemicals are often sourced from countries with lax regulations on their sale and distribution. Once the precursors are obtained, they are chemically processed to produce amphetamine, which is then combined with theophylline to create captagon tablets. The manufacturing process poses significant risks to those involved, as it involves handling hazardous chemicals and operating in unregulated conditions (Brocal et al., 2018). The quality and potency of captagon produced in illicit labs can vary, leading to potential health risks for users.

Illicit manufacturing typically entails synthesizing captagon's key components—amphetamine and theophylline—followed by the formulation of the final product (Mohaddes Ardabili et al., 2022, Wu et al., 2019).

### 4.1 Synthesis of amphetamine

Amphetamine is a key component of captagon and is synthesized through a series of organic chemistry reactions, typically involving precursors like phenylacetone (P2P) and methylamine (Schindler et al., 2021, Wu et al., 2019). According to Onoka et al. (2020), the process involves a reductive amination reaction in the primary synthesis route from P2P with *α*-benzyl*-N*-methylphenethylamine formed as a synthetic route characteristic impurity. In the reaction, the carbonyl group in P2P reacts with the amine group in methylamine to form a double bond between carbon and nitrogen, resulting in the imine. Common reducing agents used in the synthesis of amphetamine include catalytic hydrogenation (Clement et al., 2000, Yu et al., 2024). Alternative synthetic methods may employ different phenylpropane derivatives depending on precursor availability (Chambers et al., 2018). Alternative synthesis routes for amphetamine include via

1. **Leuckart reaction**, where P2P reacts with formamide or ammonium formate to produce an intermediate formamide derivative, which is subsequently hydrolyzed to amphetamine (Onoka et al., 2020, Pergolizzi et al., 2024). This method is often favored in clandestine settings due to its simplicity.
2. **Birch reduction**, which involves the reduction of phenylpropanolamine or similar precursors using alkali metals, such as sodium, in liquid ammonia (Burrows et al., 2021, Wenthur et al., 2017).

In clandestine laboratories, the synthesis is often adapted to optimize yield and purity. One of the main challenges is isolating and purifying amphetamine from by-products produced during the reactions (Rose and Söderholm, 2022). The process also involves neutralizing and extracting amphetamine base, which can then be converted into a stable salt, typically the hydrochloride form, for easier distribution and a longer shelf-life (Onoka et al., 2020).

### 4.2 Extraction of theophylline

Theophylline is another component of captagon that is typically extracted from pharmaceutical preparations containing theophylline or its derivatives, such as aminophylline (Katselou et al., 2016, Wu et al., 2019). According to the study of Monteiro et al. (2016), natural sources of theophylline, like tea leaves and cocoa (beans), contain only trace amounts, thereby making pharmaceutical products a more practical source. Extraction usually involves solvent-based methods, followed by purification steps like filtration or crystallization to isolate the active compound (Alharthy et al., 2022). In some cases, theophylline is synthetically produced through the methylation of xanthine (Singh et al., 2018). Theophylline acts at the cellular level to inhibit phosphodiesterase isoenzymes, as well as to antagonize adenosine, enhance catecholamine secretion, and modulate calcium fluxes (Vassallo and Lipsky, 1998).

### 4.3 Formulation and trafficking of captagon tablets

The reaction process for synthesizing captagon tablets involves the alkylation of theophylline using 1-bromo-2-chloroethane to form 7-(*β*-chloroethyl) theophylline. This is followed by a second reaction where amphetamine replaces the terminal halide in the 7-(*β*-chloroethyl) theophylline to create fenethylline (Rucker et al., 1987). This synthesis yields a compound that metabolizes into amphetamine and theophylline (Elliott et al., 2020). After synthesis, fenethylline is mixed with excipients and compressed into tablets using tableting equipment. These tablets may be colored, stamped, or imprinted with logos to resemble legitimate pharmaceuticals, enhancing their appeal on illicit markets (Katselou et al., 2016).

In 2021, EU Member States reported 22,000 amphetamine seizures totaling 7 tonnes. Türkiye seized 3.5 tonnes, including 13.8 million tablets labeled as "captagon" up from 2.9 million in 2020 (Logan et al., 2023). The average purity of amphetamine in European retail markets has increased by 41 % over the past decade while prices remained stable. Most illicit captagon tablets contain mostly caffeine and no amphetamine. Counterfeit captagon tablets seized in Saudi Arabia in 2021 contained 16–41 % amphetamine, significant levels of caffeine as an additive, and small amounts (<0.5 %) of methamphetamine (Logan et al., 2023). Based on a typical 170 mg tablet, this corresponds to an average amphetamine dose of 27–71 mg per tablet (Alshehri et al., 2020).

The illicit trafficking of captagon involves complex networks of supply chains, distribution channels, and smuggling routes that span across multiple countries and continents (Fong et al., 2024, Pergolizzi et al., 2024). The Middle East serves as a significant hub for the production, trafficking, and consumption of captagon, particularly in countries like Lebanon, Syria, Jordan, and Saudi Arabia (Rose, 2023). Trafficking routes in the Middle East may involve overland transportation or smuggling through maritime channels along the Mediterranean coast (Al-Imam et al., 2017). Captagon trafficking has surged in the Middle East, with Syria emerging as a global hub since its civil war began in 2011 (Steenkamp, 2024). The drug is trafficked primarily to the Arabian Peninsula, with Jordan serving as a key transit point. This trade fuels violence, destabilizes the region, and challenges counter-narcotics efforts, while advanced smuggling techniques and organized crime networks exacerbate interdiction challenges (Steenkamp, 2024, UNODC, 2024). Captagon's profitability underscores its role in sustaining conflict economies and regional instability.

Europe serves as both a destination market and a transit point for captagon trafficking (EMCDDA, 2023). Trafficking routes into Europe often originate from the Middle East, North Africa, or Eastern Europe, with captagon entering through seaports, airports, or land borders (Al-Imam et al., 2017). Traffickers may utilize sophisticated smuggling techniques, including concealment in legitimate cargo shipments or the use of couriers to transport small quantities. Regional dynamics, including porous borders, weak governance, and corruption, influence captagon trafficking routes in Africa (Steenkamp, 2024). Traffickers may exploit maritime routes along the Red Sea and Indian Ocean coastlines or use overland transportation through transnational smuggling networks (Pergolizzi et al., 2024). Africa also serves as a transit point for captagon destined for other regions, including Europe and North America, with the latter—particularly the United States—being a growing concern due to global drug trafficking networks. The global distribution of captagon involves a complex network of intermediaries, brokers, and criminal organizations operating across continents (Al-Imam et al., 2017). Captagon may be smuggled alongside other illicit drugs, such as cocaine or heroin, to diversify trafficking routes and minimize detection risks. Online darknet marketplaces and encrypted communication platforms have also emerged as channels for captagon distribution, enabling anonymous transactions and international trade (Khondakar, 2024). The production and trafficking of captagon are closely intertwined with underground economies and networks, which capitalize on the lucrative profits and strategic advantages offered by the illicit drug trade (Table 3).

**Table 3:** Illicitly produced captagon and organized crimes and terrorism.

**Region**	**Organized Crime Involvement**	**Terrorism Linkages**	**References**

Middle East	Large-scale production by organized criminal networks in countries like Syria and Lebanon, often in collaboration with corrupt officials	Significant funding source for terrorist groups, notably ISIS and Hezbollah, through production and trafficking	Al-Imam et al. (2017)
Europe	Trafficking networks dominated by Balkan and Eastern European criminal syndicates	Some financing of extremist groups, particularly through smuggling routes, but less direct involvement compared to the Middle East	UNODC (2023)
Africa	Smuggling facilitated by transnational crime groups across North Africa and the Sahel regions	Africa serves primarily as a transit route, with some funds potentially channeled to terrorist organizations, especially in Libya and Mali	UNODC (2023)
Asia	Production and trafficking by criminal organizations in regions such as South and Southeast Asia	Captagon trade is linked to insurgency financing, particularly for groups like the Taliban in Afghanistan and other local militant groups	Reid et al. (2006)
Online (Darknet)	Sale of captagon facilitated by underground online markets, with the anonymity provided to sellers and buyers	Darknet platforms may serve to fund extremist or terrorist organizations, but the scale is less clear than in other regions	Al-Imam et al. (2017)

### 4.4 Organized crime

Transnational criminal groups, including organized unlawful syndicates and drug cartels, play a central role in the production and trafficking of captagon (Rose, 2023). These criminal organizations control various aspects of the drug trade, from precursor chemical sourcing and laboratory operations to distribution networks and money laundering. The profitability of captagon fuels violent competition among rival criminal groups, leading to turf wars, assassinations, and other forms of violence (Al-Imam et al., 2017).

### 4.5 Terrorism financing

The production and trafficking of captagon have been linked to terrorism financing, particularly in conflict-affected regions like Syria and Iraq (Wu et al., 2019). Terrorist organizations, including ISIS and Hezbollah, have reportedly profited from the illicit captagon trade to fund their activities, purchase weapons, and recruit fighters (Steenkamp, 2024). The anonymity and profitability of the drug trade provide terrorist groups with a steady source of revenue, enabling them to sustain their operations and expand their influence.

### 4.6 State sponsorship

Some state actors have been implicated in facilitating the production and trafficking of captagon as a means of exerting influence, funding proxy militias, or destabilizing rival governments (Al-Imam et al., 2017, Mohaddes Ardabili et al., 2022). State-sponsored trafficking networks may benefit from official complicity, diplomatic immunity, or state resources to evade law enforcement and carry out illicit activities with impunity.

## 5 Social and economic impacts of captagon

The captagon trade poses substantial challenges for regional and extra-regional rule of law, security, political stability, and public health. The European Union, the United States, and the United Kingdom now perceive it as a geopolitical challenge affecting their interests both domestically and in the Middle East (Malik and Gallien, 2019, Steenkamp, 2024). The situation is further complicated by the long-term position of the EMR as one of the world's largest opium production hubs, hindering the provision of adequate health and social care services (Rostam-Abadi et al., 2023). In 2017, approximately 4.2 million disability-adjusted life years (DALYs) were lost to substance use disorders. The instability of regions marked by war, insurgency, political conflict, and civil unrest profoundly affects substance use, impacting production, trade, availability, and patterns of use (WHO, 2019).

### 5.1 Disruption of communities

Despite captagon's disadvantage in Europe's illicit markets, its broad appeal warrants attention. Authorities should not dismiss its potential to exacerbate challenges posed by synthetic drugs (Rose, 2023, Schifano et al., 2023).

The increasing trend of clandestine manufacture and use of amphetamine-type stimulants has become a significant concern in countries such as Iran, Morocco, and Pakistan. There is also a high demand for fenethylline tablets in some countries of the region, especially in Syria, Lebanon, and countries in the Arabian Peninsula (Herbert, 2017, Mohaddes Ardabili et al., 2022). Authorities in Saudi Arabia, Kuwait, and Qatar report that the use of fenethylline is prevalent among their younger, affluent citizens (Katselou et al., 2016). In Arab countries, millions of captagon tablets are seized every year, representing one-third of global amphetamine seizures within a year. Authorities in Saudi Arabia announced the seizure of 100 million captagon tablets within the first five months of 2022. According to the United Nations Office on Drugs and Crime (UNODC), the three countries reporting the highest captagon seizures are Saudi Arabia, Jordan, and Syria (Wazaify et al., 2024). However, data on its supply, use, and harm remains largely anecdotal (Ghiabi, 2018, Wazaify et al., 2024). In Saudi Arabia, there are more hospitalizations due to captagon use than opioid use (Alkhalaf et al., 2019).

### 5.2 Economic consequences

Economies confront substantial productivity losses attributed to drug-related challenges, where the economic burden, encompassing treatment expenses, diminished work capacity, and heightened security costs, weighs significantly on individual countries (Tumalavicius, 2023). Emerging regional substance use trends, influenced by cultural expectations and the displacement of large populations, find exemplification in the case of captagon (Ghiabi, 2018). The captagon trade, estimated at generating an alternative revenue of $2.7 billion annually for the Syrian regime and its partners (Hezbollah and Iran-aligned militias), undermines the interests of the EU and its regional partners (Herbert, 2017, Rose, 2023). Responding to these challenges, the EU has initiated a counter-captagon strategy, imposing sanctions on major players connected to the Assad regime and Hezbollah. This collaborative effort aligns with joint sanctions by the US and the UK (Rose, 2023). The EU aims to bolster intelligence exchange and adopt best practices to mitigate the supply and demand for captagon. While monitoring the trade and implementing sanctions, the absence of a comprehensive counter-captagon strategy and multilateral framework within EU policy necessitates urgent establishment (Levallois et al., 2023). It is imperative for the EU to develop a parallel inter-agency strategy, enhance public awareness of captagon's health implications, and foster collaboration with Western and Middle Eastern partners, establishing a captagon-specific multilateral mechanism focused on information exchange, dialogue, interdiction, and harm-reduction strategies (Ghiabi, 2018, Rose, 2023, Soderholm, 2017).

### 5.3 Regulatory and legislative landscape of captagon

The ongoing captagon epidemic has prompted various countries, particularly in the Middle East, to respond with legislative measures aimed at controlling its production, distribution, and use. At the national level, various countries have implemented strict regulations to combat the trafficking and misuse of captagon. For instance, countries such as Saudi Arabia, Lebanon, and Jordan have implemented stringent laws to address the trafficking and misuse of captagon (Al-Imam et al., 2017). Saudi Arabia has taken significant steps to classify captagon as a controlled substance, thereby criminalizing its production and distribution. The Saudi government has established a comprehensive legal framework that includes severe penalties for trafficking and possession, reflecting the drug's association with organized crime and terrorism financing (Tobaiqy and Al-Asmari, 2024). These measures are part of broader efforts to address the public health crisis associated with captagon misuse, which has been linked to increased rates of addiction and related health issues. The Lebanese government has increased its law enforcement efforts to combat the drug trade, including cross-border cooperation with neighboring countries to disrupt trafficking networks. However, the effectiveness of these measures is often hampered by corruption and the complex socio-political landscape in the region, which can undermine enforcement efforts (Katselou et al., 2016). In Jordan, the government has recognized the growing issue of captagon misuse and has initiated programs aimed at prevention and treatment. Legislative measures have been introduced to enhance the capacity of healthcare systems to address substance use disorders, including the establishment of rehabilitation centers specifically for captagon users (Katselou et al., 2016).

Internationally, there is a growing recognition of the need for coordinated legislative efforts to combat the transnational nature of captagon trafficking. The United Nations Office on Drugs and Crime (UNODC) has emphasized the importance of international cooperation in enforcing drug laws and sharing intelligence among countries (Heikkilä et al., 2021, UNODC, 2023). This includes the development of treaties and agreements that facilitate the monitoring of precursor chemicals used in the synthesis of captagon, thereby aiming to curb its illicit production (Heikkilä et al., 2021, Negrei et al., 2017). One significant aspect of U.S. legislative attempts to control captagon involves its classification under the Controlled Substances Act. Currently, fenethylline is not explicitly listed as a controlled substance at the federal level, which complicates enforcement efforts against its trafficking and misuse (Katselou et al., 2016). The lack of specific legislation targeting captagon means that law enforcement agencies often rely on broader drug trafficking laws that apply to amphetamines and other stimulants. This gap in legislation can hinder efforts to address the unique challenges posed by captagon, especially as its use becomes more prevalent in certain communities. Some experts argue that the inclusion of captagon in the list of controlled substances would facilitate stricter penalties for its production and distribution (Al-Imam et al., 2017). This is particularly relevant given the drug's association with organized crime and its reported use by militant groups in conflict zones, which has implications for national security (Al-Imam et al., 2017). Some states in the United States have begun to implement educational programs aimed at raising awareness about the dangers of captagon and other stimulants, targeting at-risk populations such as youth and military personnel (Al Omari et al., 2022). These initiatives are part of a broader strategy to mitigate substance misuse through prevention and education rather than solely relying on punitive measures.

## 6 Recommendations and conclusion

The psychostimulant drug captagon, originally developed for medical use, has evolved into a significant public health and security threat. The drug's clandestine production, particularly in the Middle East, has led to widespread misuse, contributing to serious health risks, including addiction, cardiovascular complications, and amphetamine-induced psychosis. Despite its classification as a Schedule II substance under the United Nations Convention on Psychotropic Substances, captagon continues to be produced illegally, with counterfeit versions dominating the market. These tablets often contain mixtures of harmful substances, posing unpredictable risks to users. Key drivers of the illicit use of captagon include economic and social factors, particularly in regions affected by conflict, where the drug is used as both a stimulant for endurance and as a tool for financing unlawful syndicates. The complexity of its production and trafficking networks, combined with the drug's affordability and accessibility, has enabled its widespread use, particularly among youth and vulnerable populations. Additionally, captagon's involvement in organized underground economies and unlawful syndicate financing exacerbates instability in already fragile regions, presenting geopolitical challenges for the global community.

Global collaboration between governments, law enforcement agencies, and international organizations is critical to disrupt the production, trafficking, and distribution of captagon. Efforts could focus on intelligence sharing, coordinated enforcement actions, and stricter regulation of precursor chemicals used in captagon production. Establishing a multilateral framework dedicated to counter-captagon operations could enhance the effectiveness of such efforts. Governments, particularly in high-prevalence regions like the Middle East, could prioritize harm reduction strategies, including public education campaigns, accessible treatment programs for addiction, and comprehensive rehabilitation services. Increased awareness of the health risks associated with captagon, particularly among vulnerable groups such as youth and displaced populations, is essential for preventing misuse. Tackling the underlying socioeconomic factors contributing to the illicit use of captagon is essential. Economic instability, unemployment, and lack of access to education create environments where illicit use of drugthrives. Investing in community development, job creation, and social services can help mitigate the demand for illicit drugs. The link between captagon trafficking and terrorism financing necessitates a comprehensive approach that integrates counter-terrorism efforts with drug enforcement. Targeting the financial networks of terrorist organizations involved in the captagon trade is vital for disrupting their operations and reducing their influence.

Continued research is essential to develop evidence-based treatment protocols and effective law enforcement strategies. To advance understanding of fenethylline’s synthesis, use, metabolism, and pharmacokinetics, the following actions are recommended:

1. Facilitate collaborations between law enforcement and researchers to analyze real-world seized samples under controlled conditions.
2. Foster cross-border collaborations to share expertise, data, and resources.
3. Develop computational and in vitro models to study fenethylline metabolism and effects without requiring large quantities of the drug.
4. Prioritize grants for controlled studies on fenethylline’s pharmacology and societal impacts.
5. Create centralized databases to promote transparency and accelerate progress in understanding fenethylline’s properties.

These steps will bridge knowledge gaps, enabling better scientific insights and informed policy-making. Addressing the captagon crisis requires a comprehensive, multi-faceted approach involving international cooperation, public health initiatives, socioeconomic reform, and continued research. Only through sustained efforts at all levels can the global community hope to curb the production, trafficking, and misuse of thispsychostimulant.

## Ethical responsibilities of authors

All authors have read, understood, and have complied as applicable with the statement on "Ethical responsibilities of Authors" as found in the Instructions for Authors

## Ethics approval and consent to participate

Not applicable

## Consent for publication

All the author expressly consents to the publication of the work.

## Funding

The contributions of co-authors from CZU was supported by the METROFOOD-CZ research infrastructure project (MEYS Grant No: LM2023064), which includes access to its facilities.

## Author’s contribution

JP conceived and designed the work; All the authors contributed to the literature review while MO and MM developed the first manuscript draft. Every author took turns to correct and revise it while MM and PT wrote the final manuscript draft. All the authors contributed to and approved the final submitted version.

## CRediT authorship contribution statement

**Patočka Jiří:** Writing – review & editing, Writing – original draft, Visualization, Validation, Supervision, Software, Resources, Project administration, Methodology, Investigation, Formal analysis, Data curation, Conceptualization. **Tlustoš Pavel:** Writing – review & editing, Writing – original draft, Visualization, Validation, Supervision, Software, Resources, Project administration, Methodology, Investigation, Formal analysis, Data curation, Conceptualization. **Malík Matěj:** Writing – review & editing, Writing – original draft, Visualization, Validation, Supervision, Software, Resources, Project administration, Methodology, Investigation, Formal analysis, Data curation, Conceptualization. **Ogwu Matthew:** Writing – review & editing, Writing – original draft, Visualization, Validation, Supervision, Software, Resources, Project administration, Methodology, Investigation, Funding acquisition, Formal analysis, Data curation, Conceptualization.

## Declaration of Competing Interest

The authors declare that they have no known competing financial interests or personal relationships that could have appeared to influence the work reported in this paper.

## References

[bib1] Abazid H., Patel V.B., Preedy V.R. (2022). Handbook of Substance Misuse and Addictions.

[bib2] Abu-Hashem A.A., Hakami O., El-Shazly M., El-Nashar H.A.S., Yousif M.N.M. (2024). Caffeine and purine derivatives: a comprehensive review on the chemistry, biosynthetic pathways, synthesis-related reactions, biomedical prospectives and clinical applications. Chem. Biodivers..

[bib3] Adinoff B. (2004). Neurobiologic processes in drug reward and addiction. Harv. Rev. Psychiatry.

[bib4] Al Omari O., Wynaden D., Alkhawaldeh A., Alhalaiqa F., Al Dameery K., Roach E.J., Sunderraj S.J., Khalaf A. (2022). Jordanian University Students' lived experience of misusing amphetamine (captagon): a qualitative study. J. Addict. Nurs..

[bib5] Alabdalla M.A. (2005). Chemical characterization of counterfeit captagon tablets seized in Jordan. Forensic Sci. Int..

[bib6] Alabdulla M., Samarasinghe N., Tulley I., Reagu S. (2022). Evolution of policy for the treatment of substance use disorders in Qatar. Subst. Abus. Treat. Prev. Policy.

[bib7] Alghamdi M., Alqahtani B., Alhowti S. (2016). Cardiovascular complications among individuals with amphetamine-positive urine drug screening admitted to a tertiary care hospital in Riyadh. J. Saudi Heart Assoc..

[bib8] Alharthy S.A., Alharthi M.A., Almalki S.A., Alosaimi S.H., Aqeel A.H., Altowairqi S.A., Alsalahat I., Panda D.S., Alsheikh M.Y., Naguib I.A. (2022). Green assessment of chromatographic methods used for the analysis of four methamphetamine combinations with commonly abused drugs. Separations.

[bib9] Al-Imam A., AbdulMajeed B.A. (2017). Captagon, octodrine, and nbome: an integrative analysis of trends databases, the deep web, and the darknet. Glob. J. Health Sci..

[bib10] Al-Imam A., Santacroce R., Roman-Urrestarazu A., Chilcott R., Bersani G., Martinotti G., Corazza O. (2017). Captagon: use and trade in the Middle East. Hum. Psychopharmacol. Clin. Exp..

[bib11] Alkhalaf A., Alahmari T., Ashworth F. (2019). Changing trends of substance addiction in Saudi Arabia between 1993 and 2013. MOJ Addict. Med. Ther..

[bib12] Al-Obaidi T.A., Fletcher S.M. (2014). Management of clandestine drug laboratories: need for evidence-based environmental health policies. Environ. Health Prev. Med..

[bib13] Alshehri A.Z., Al Qahtani Ms, Al Qahtani M.A., Faeq A.M., Aljohani J., Alfarga A. (2020). GC-MS analysis of adulterants in captagon tablet. J. Chem. Nutr. Biochem.

[bib14] Alshenguity A., Aloufi K., Alkurdi A., Aljabri D., Almohammadi M., Altamimi T., Alshenguity A., Alhelali H., Alharbi A., Aljihani S. (2023). Socio-Demographic correlates and patterns of use among patients visiting addiction services of almadinah specialized psychiatric hospital in Saudi Arabia. J. Health Sci..

[bib15] Arnaud M.J. (2011).

[bib16] Bamofleh E.A., Mohammed J.A., Abdelrahim M.E.A., Gamal M. (2017). The reasons behind prevalence of captagon addiction in Jeddah and community awareness: a Questionnaire-based study. Sch. Rep..

[bib17] Bollu P.C., Kaur H. (2019). Sleep medicine: insomnia and sleep. Mo. Med..

[bib18] Brocal F., Gonzalez C., Reniers G., Cozzani V., Sebastian M.A. (2018). Risk management of hazardous materials in manufacturing processes: links and transitional spaces between occupational accidents and major accidents. Materials.

[bib19] Burrows J., Kamo S., Koide K. (2021). Scalable Birch reduction with lithium and ethylenediamine in tetrahydrofuran. Science.

[bib20] Caesar E. (2024). The New Yorker. How Syr. became Middle East'S. Drug Deal..

[bib21] Castells X., Blanco-Silvente L., Cunill R. (2018). Amphetamines for attention deficit hyperactivity disorder (ADHD) in adults. Cochrane Database Syst. Rev..

[bib22] Chambers S.A., DeSousa J.M., Huseman E.D., Townsend S.D. (2018). The dark side of total synthesis: strategies and tactics in psychoactive drug production. ACS Chem. Neurosci..

[bib23] Citaristi I. (2022). The Europa Directory of International Organizations 2022.

[bib24] Ciucă Anghel D.-M., Nițescu G.V., Tiron A.-T., Guțu C.M., Baconi D.L. (2023). Understanding the mechanisms of action and effects of drugs of abuse. Molecules.

[bib25] Clement B., Behrens D., Möller W., Cashman J.R. (2000). Reduction of amphetamine hydroxylamine and other aliphatic hydroxylamines by benzamidoxime reductase and human liver microsomes. Chem. Res. Toxicol..

[bib26] Costa V.M., Grando L.G.R., Milandri E., Nardi J., Teixeira P., Mladenka P., Remiao F., On Behalf Of The, O (2022). Natural sympathomimetic drugs: from pharmacology to toxicology. Biomolecules.

[bib27] Dabbagh R., Rawson R. (2019). Captagon Use in Saudi Arabia: What Do we Know?. Int. Addict. Rev..

[bib28] Desoky E.S.E.L., El-Tantawy A.M.A., Raya Y.M., Al-Yahya A. (2011). Amphetamine versus non amphetamine-related first episode psychosis in Saudi Arabian Patients. Pharm Pharm.

[bib29] Di Giovanni S., Varriale A., Marzullo V.M., Ruggiero G., Staiano M., Secchi A., Pierno L., Fiorello A.M., D'Auria S. (2012). Determination of benzyl methyl ketone – a commonly used precursor in amphetamine manufacture. Anal. Methods.

[bib30] Docherty J.R., Alsufyani H.A. (2021). Pharmacology of drugs used as stimulants. J. Clin. Pharmacol..

[bib31] El Khoury J. (2018). The use of stimulants in the ranks of Islamic State: myth or reality of the syrian conflict. Stud. Confl. Terror..

[bib32] Elasfar A.A., Ahmad K.E., AlShaghaa W. (2014). Clinical characteristics and outcome of heart failure and captagon amphetamine use: an observational prospective study. Egypt. Heart J..

[bib33] Elliott S.P., Holdbrook T., Brandt S.D. (2020). Prodrugs of new psychoactive substances (NPS): a new challenge. J. Forensic Sci..

[bib34] Ellison T., Levy L., Bolger J.W., Okun R. (1970). The metabolic fate of ^3^H-fenetylline in man. Eur. J. Pharmacol..

[bib35] EMCDDA (2018). Publications Office.

[bib36] EMCDDA (2023). Lisbon.

[bib37] Faraone S.V. (2018). The pharmacology of amphetamine and methylphenidate: Relevance to the neurobiology of attention-deficit/hyperactivity disorder and other psychiatric comorbidities. Neurosci. Biobehav. Rev..

[bib38] Feltenstein M.W., See R.E. (2008). The neurocircuitry of addiction: an overview. Br. J. Pharmacol..

[bib39] Ferrucci M., Limanaqi F., Ryskalin L., Biagioni F., Busceti C.L., Fornai F. (2019). The effects of amphetamine and methamphetamine on the release of norepinephrine, dopamine and acetylcholine from the brainstem reticular formation. Front. Neuroanat..

[bib40] Fong S., Carollo A., Rossato A., Prevete E., Esposito G., Corazza O. (2024). Captagon: A comprehensive bibliometric analysis (1962-2024) of its global impact, health and mortality risks. Saudi Pharm. J..

[bib41] Ghiabi M., Klein A., Stothard B. (2018). Collapse of the Global Order on Drugs: From UNGASS 2016 to Review 2019.

[bib42] Goenechea S., Brzezinka H. (1984). Detection and identification of a new metabolite of fenethylline. Arch. Kriminol..

[bib43] Goh J.W., Thaw M.M., Ramim J.U., Mukherjee R. (2023). Theophylline toxicity: a differential to consider in patients on long-term theophylline presenting with nonspecific symptoms. Cureus.

[bib44] Gokdemir M.T., Giden R. (2019). Acute inferior myocardial infarction associated with the ingestion of captagon pills: a case report. Turk. J. Emerg. Med..

[bib45] Gordan R., Gwathmey J.K., Xie L.H. (2015). Autonomic and endocrine control of cardiovascular function. World J. Cardiol..

[bib46] Hamdan M., El Hayek S., Bizri M. (2021). Captagon use in a patient with iatrogenic opioid use disorder. Curr. Psychopharmacol..

[bib47] Heal D.J., Smith S.L., Gosden J., Nutt D.J. (2013). Amphetamine, past and present--a pharmacological and clinical perspective. J. Psychopharmacol..

[bib48] Heikkilä H., Maalouf W., Campello G. (2021). The United Nations office on drugs and crime’s efforts to strengthen a culture of prevention in low- and middle-income countries. Prev. Sci..

[bib49] Herbert M. (2017). Middle east drugs bazaar: production, prevention, and consumption. Bustan Middle East Book Rev..

[bib50] Janitschke D., Lauer A.A., Bachmann C.M., Seyfried M., Grimm H.S., Hartmann T., Grimm M.O.W. (2020). Unique role of caffeine compared to other methylxanthines (theobromine, theophylline, pentoxifylline, propentofylline) in regulation of AD relevant genes in neuroblastoma SH-SY5Y wild type cells. Int. J. Mol. Sci..

[bib51] Jilani T.N., Preuss C.V., Sharma S. (2023). Theophylline.

[bib52] Kaleta E., Ketha H., Garg U. (2020). Toxicology Cases for the Clinical and Forensic Laboratory.

[bib53] Katselou M., Papoutsis I., Nikolaou P., Qammaz S., Spiliopoulou C., Athanaselis S. (2016). Fenethylline (Captagon) abuse - local problems from an old drug become universal. Basic Clin. Pharmacol. Toxicol..

[bib54] Katz S.M. (2022). Captagon War: Violent N. Drug Trade Setting Middle East Fire. Men. 'S. J..

[bib55] Keefe P.R. (2016). The Drugs of War—Captagon and the Islamic State, Report Criminal Justice.

[bib56] Khalil M., Tabar N.A., Alsadi M., Khrais H., Oweidat I., Hamaideh S.H., Hamdan-Mansour A.M. (2022). Factors associated with substance use disorder: male adolescents’ lived experience. Int. J. Ment. Health Addict..

[bib57] Khondakar H.K. (2024). Golden triangle to Bangladesh: regional analysis of ats sources, trafficking routes and kingpin involved, and role of regional organizations. Int. Res. Soc. Sci..

[bib58] Kravitz M., Nichols W. (2016). A bitter pill to swallow: connections between captagon, Syria, and the Gulf. J. Int. Aff..

[bib59] Kristen G., Schaefer A., Von Schlichtegroll A. (1986). Fenetylline: Therapeutic use, misuse and/or abuse. Drug Alcohol Depend..

[bib60] Laniel L. (2017). Captagon: déconstruction d’un mythe. OFDT.

[bib61] Levallois A., Kasapoğlu C., Tür Ö., Dalay G. (2023).

[bib62] Logan, B.K., Mohr, A.L.A., Browne, T., 2023. Colombo Plan Health Alert Counterfeit Captagon. Colombo Plan via U.S. Department of State/INL. 〈https://www.cfsre.org/images/content/reports/public_alerts/CAPTAGON_Public_Health_Alert_FINAL.pdf〉 (accessed 6 February 2025).

[bib63] Lucchetti M., Kaminska M., Oluwasegun A.K., Mosig A.S., Wilmes P. (2021). Emulating the gut-liver axis: dissecting the microbiome's effect on drug metabolism using multiorgan-on-chip models. Curr. Opin. Endocr. Metab. Res..

[bib64] Malik A., Gallien M. (2019). Border economies of the Middle East: why do they matter for political economy?. Rev. Int. Political Econ..

[bib65] Martin, D., Le, J.K., 2023. Amphetamine. StatPearls Publishing, Treasure Island (FL). 32310563

[bib66] Mathot S. (2018). Pupillometry: psychology, physiology, and function. J. Cogn..

[bib67] Mohaddes Ardabili H., Akbari A., Rafei P., Butner J.L., Khan R., Khazaal Y., Arab A.Z., Qazizada M.R., Al-Ansari B., Baldacchino A.M. (2022). Tramadol, captagon and khat use in the Eastern Mediterranean Region: opening pandora's box. BJPsych Int.

[bib68] Monteiro J.P., Alves M.G., Oliveira P.F., Silva B.M. (2016). Structure-bioactivity relationships of methylxanthines: trying to make sense of all the promises and the drawbacks. Molecules.

[bib69] Murphy C. (2012). Wilson center. A Kingd. 'S. Future.: Saudi Arab. eyes its Twenty.

[bib70] Negrei C., Galateanu B., Stan M., Balalau C., Dumitru M.L.B., Ozcagli E., Fenga C., Kovatsi L., Fragou D., Tsatsakis A. (2017). Worldwide legislative challenges related to psychoactive drugs. DARU J. Pharm. Sci..

[bib71] Nieddu M., Baralla E., Sodano F., Boatto G. (2023). Analysis of 2,5-dimethoxy-amphetamines and 2,5-dimethoxy-phenethylamines aiming their determination in biological matrices: a review. Forensic Toxicol..

[bib72] Onoka I., Banyika A.T., Banerjee P.N., Makangara J.J., Dujourdy L. (2020). A review of the newly identified impurity profiles in methamphetamine seizures. Forensic Sci. Int. Synerg..

[bib73] Pergolizzi J., LeQuang J.A.K., Vortsman E., Magnusson P., El-Tallawy S.N., Wagner M., Salah R., Varrassi G. (2024). The emergence of the old drug captagon as a new illicit drug: a narrative review. Cureus.

[bib74] Pires B., Rosendo L.M., Brinca A.T., Simao A.Y., Barroso M., Rosado T., Gallardo E. (2023). The therapeutic potential of amphetamine-like psychostimulants. Life.

[bib75] Reid G., Devaney M.L., Baldwin S. (2006). ASIA PACIFIC COLUMN: drug production, trafficking and trade in Asia and pacific island countries. Drug Alcohol Rev..

[bib76] Rose C. (2023). The reach of the trade in Captagon beyond the Middle East. Eur. View..

[bib77] Rose, C., Söderholm, A., 2022. The Captagon Threat: A Profile of Illicit Trade, Consumption, and Regional Realities. New Lines Institute. 〈https://newlinesinstitute.org/state-resilience-fragility/illicit-economies/the-captagon-threat-a-profile-of-illicit-trade-consumption-and-regional-realities/〉 (accessed 6 February 2025).

[bib78] Rostam-Abadi Y., Gholami J., Jobehdar M.M., Ardeshir M., Aghaei A.M., Olamazadeh S., Taj M., Saeed K., Mojtabai R., Rahimi-Movaghar A. (2023). Drug use, drug use disorders, and treatment services in the Eastern Mediterranean region: a systematic review. Lancet Psychiatry.

[bib79] Rothman R.B., Baumann M.H., Dersch C.M., Romero D.V., Rice K.C., Carroll F.I., Partilla J.S. (2001). Amphetamine-type central nervous system stimulants release norepinephrine more potently than they release dopamine and serotonin. Synapse.

[bib80] Rucker G., Neugebauer M., Neugebauer M., Heiden P.G. (1987). The chemical stability of fenethylline. Arch. Pharm..

[bib81] Rusyniak D.E. (2011). Neurologic manifestations of chronic methamphetamine abuse. Neurol. Clin..

[bib82] Schifano F., Vento A., Scherbaum N., Guirguis A. (2023). Stimulant and hallucinogenic novel psychoactive substances; an update. Expert Rev. Clin. Pharmacol..

[bib83] Schindler C.W., Thorndike E.B., Partilla J.S., Rice K.C., Baumann M.H. (2021). Amphetamine-like neurochemical and cardiovascular effects of *α*-ethylphenethylamine analogs found in dietary supplements. J. Pharmacol. Exp. Ther..

[bib84] Sevarino K.A., Farrell M., Tasman A., Riba M.B., Alarcón R.D., Alfonso C.A., Kanba S., Lecic-Tosevski D., Ndetei D.M., Ng C.H., Schulze T.G. (2023). Tasman’s Psychiatry.

[bib85] Singh N., Shreshtha A.K., Thakur M.S., Patra S. (2018). Xanthine scaffold: scope and potential in drug development. Heliyon.

[bib86] Soderholm A., Corazza O., Roman-Urrestarazu A. (2017). Novel Psychoactive Substances.

[bib87] Sofuoglu M., Sewell R.A. (2009). Norepinephrine and stimulant addiction. Addict. Biol..

[bib88] Steenkamp C. (2024). Captagon and conflict: drugs and war on the border between Jordan and Syria. Mediterr. Polit..

[bib89] Thomas J. (2013). in the Gulf States.

[bib90] Tobaiqy M., Al-Asmari A.I. (2024). Substance misuse disorder in Saudi Arabia: a comprehensive examination of current demographic patterns, trends, and intervention requirements. Saudi Pharm. J..

[bib91] Tumalavicius V. (2023). Ensuring the secure and sustainable development of the society: countering drug trafficking at the global level. Access J. Access Sci. Bus. Innov. Digit. Econ..

[bib92] Ulucay A., Arpacik Kargi C., Aksoy M.F. (2012). Acute myocardial infarction associated with Captagon use. Anadolu Kardiyol. Derg..

[bib93] UNODC (United Nations Office on Drugs and Crime), 2023. World Drug Report. United Nations. Vienna. p. 202. 〈https://www.unodc.org/res/WDR-2023/WDR23_Booklet_2.pdf〉.

[bib94] UNODC (United Nations Office on Drugs and Crime), 2024. Drug Trafficking Dynamics across Iraq and the Middle East (2019–2023): Trends and Responses. United Nations. p. 45. 〈https://www.unodc.org/documents/data-and-analysis/Iraq/Iraq_regional_dynamics_report_2024.pdf〉.

[bib95] Vassallo R., Lipsky J.J. (1998). Theophylline: recent advances in the understanding of its mode of action and uses in clinical practice. Mayo Clin. Proc..

[bib96] Wagner D.J., Sager J.E., Duan H., Isoherranen N., Wang J. (2017). Interaction and transport of methamphetamine and its primary metabolites by organic cation and multidrug and toxin extrusion transporters. Drug Metab. Dispos..

[bib97] Wazaify M., Al-Khateeb Y.M., Musleh B., Al-Smadi H., Steenkamp C. (2024). Qualitative exploration of the experiences of people who use captagon and therapists in Jordan. Subst. Use Misuse.

[bib98] Wenthur C.J., Zhou B., Janda K.D. (2017). Vaccine-driven pharmacodynamic dissection and mitigation of fenethylline psychoactivity. Nature.

[bib99] White C.M., Browne T., Nafziger A.N. (2021). Inherent dangers of using non-us food and drug administration-approved substances of abuse. J. Clin. Pharmacol..

[bib100] WHO (World Health Organization) (2019). Regional framework for action to strengthen the public health response to substance use. Reg. Off. East. Mediterr..

[bib101] Wu N., Feng Z., He X., Kwon W., Wang J., Xie X.Q. (2019). Insight of captagon abuse by chemogenomics knowledgebase-guided systems pharmacology target mapping analyses. Sci. Rep..

[bib102] Yasin H., Bulatova N., Wazaify M. (2020). Patterns of substance use among patients in addiction rehabilitation in Jordan. Subst. Use Misuse.

[bib103] Yoshimura H., Yoshimitsu T., Yamada H., Koga N., Oguri K. (1988). Metabolic fate of fenetylline in rat and man. Xenobiotica.

[bib104] Yu M., Ouyang D., Wang L., Liu Y.-N. (2024). Catalytic reduction of aromatic nitro compounds to phenylhydroxylamine and its derivatives. Molecules.

[bib105] Zhao M., Ma J., Li M., Zhang Y., Jiang B., Zhao X., Huai C., Shen L., Zhang N., He L., Qin S. (2021). Cytochrome P450 enzymes and drug metabolism in humans. Int. J. Mol. Sci..

